# ZenBand: a numerical solver of photonic crystals with a graphical user interface

**DOI:** 10.1038/s41598-026-37129-2

**Published:** 2026-02-04

**Authors:** Andrius Zinkevičius, Ignas Lukošiūnas, Darius Gailevičius

**Affiliations:** Laser Research Center, Faculty of Physics, Vilnius University, Saulėtekio Ave. 10, Vilnius, Lithuania

**Keywords:** Optics and photonics, Physics

## Abstract

We developed an open-source Plane Wave Expansion Method solver using Python and a custom Tkinter library to solve a design oriented problem of photonic crystal dispersion for known classical examples, custom geometries, and symmetries. Such structures are capable of light confinement, omnidirectional reflection, beam collimation and negative refraction. We dive deeper into the diagonally anisotropic photonic crystals, whose Plane Wave Expansion algorithm is directly embedded in the application. The user interface is present in the developer’s repository link: https://github.com/ZenTunturi/ZenBand.

## Introduction

The necessity of open-source numerical software in modern times is of significant importance. Especially open-access solvers that aid in solving differential equations numerically for conservative and non-conservative optical systems^1–3^, as well as for data analysis^4–6^ and image processing^7–9^. The main difficulty with preexisting commercial software, such as Lumerical, is the high license pricing, data analysis limitations, numerical problem-solving specialization, and extensive tutorial requirements due to the software’s complexity. Such limitations, with respect to the developers of the commercial software, delay timely progress in photonic system simulations and system modeling for system designers and engineers.

Nowadays, there are free-to-use alternatives for many widely studied algorithms that have many built-in optimization tools. For example, Meep^10^ is a C++ library that simplifies Finite Difference Time Domain (FDTD) simulations by providing simple and intuitive functions. These functions enable users to create a simulation window with only a fraction of the lines of code. Also, the Rigorous Coupled Wave Analysis (RCWA) algorithm is implemented in TORCWA^11^ as well as MAXIM^12^ software for periodically distributed linear optical materials. The former is a GPU-accelerated Python library with integrated geometric parameter optimization, utilizing Python libraries for automatic differentiation when calculating the gradient of the evaluation function. MAXIM is more suited for users who are not as familiar with programming, as it features a highly intuitive graphical user interface (GUI).

If a numerical model of a counter-top optical system is needed, the FDTD method can appear to be too tedious. A great alternative for this need is presented by “Diffractio”^13^—an open-source Python package that simplifies the analysis of diffraction and interference, both within the scalar and vector optics approaches. This library is intuitively oriented around three modules: sources, masks and fields.

**Fig. 1:**
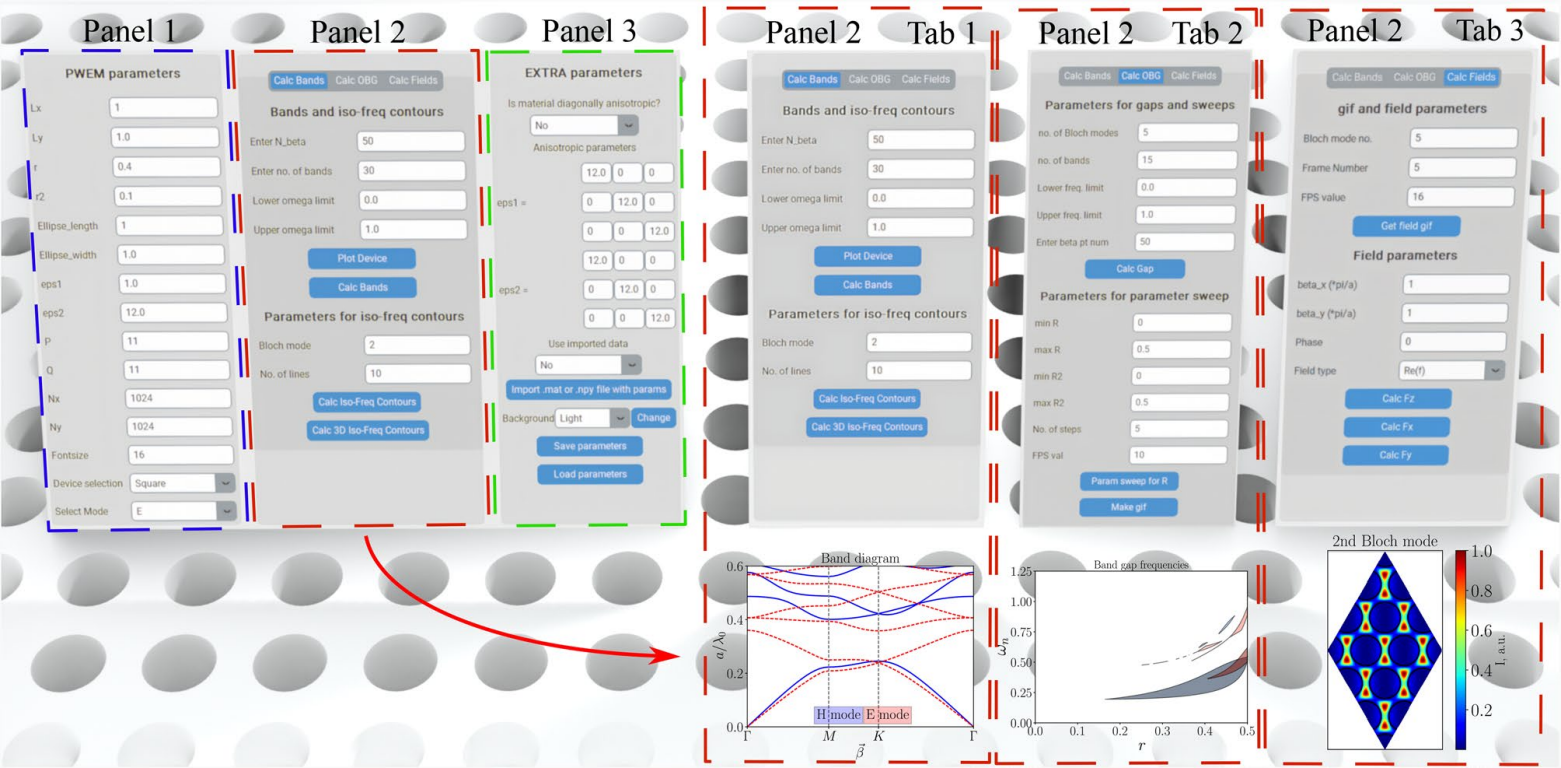
Application layout scheme for photonic crystal computations. The UI consists of three panels. Panel 1 holds the general parameters, Panel 2 holds the functions and Panel 3 holds additional features. The second panel is made of three tabs - Tab 1 serves band and iso-frequency calculations for generalized symmetries, Tab 2 is for omnidirectional band-gap analysis and Tab 3 is for field calculations.

Another field of photonics that numerical software has helped to improve is the fabrication of diffractive optical elements (DOEs). MetaOptics^14^ and GDOESII^15^ are such applications with simple GUIs, that help users create metasurface phase-masks using simple meta-atom geometries.

Numerous numerical software packages have been developed for various specialized fields of optics. BMP-Matlab^16^ is an advanced, computationally efficient algorithm that provides multi-mode analysis through a wide variety of fiber geometries and even accounts for the effect of bends. In addition, PyWolf^17^ is a software that computes the propagation of partially coherent light by examining the evolution of a cross-spectral density function of two-dimensional light sources in both near-field and far-field approximations. The software exhibits excellent performance, as the 4D cross-spectral density array elements can be computed in parallel. PtyLab^18^ is an open-source ptychographic reconstruction software, that was made with accessibility in mind. The software is multi-lingual—it is available in MATLAB, Python, and Julia.

The progress of CPU-based open-source libraries (such as numpy, scipy, matplotlib, pyvista, and others) shows excellent results in constructing eigenvalue-based algorithm libraries that are applied for electromagnetic simulations^19–22^. Concurrently, suppose the array dimensions are large enough. In that case, various solver libraries are well-written and developed for open-source usage, such as 3D RCWA-based Torcwa, which utilizes PyTorch as its primary library for numerical calculations and analysis of periodic structures. Recent advancements are being made for photonic crystal slabs, which involve the use of libraries for dispersion calculation for 2D photonic crystals^23–26^.

However, with the intense and successful progress being made in open-source library additions, there is a relatively small amount of open software that utilizes these libraries for User Interface (UI) construction. In fact, open-source libraries can be combined with one another to form an open-source software, which in turn provides simplicity in the use of libraries for numerical modeling. Specifically, using PyQt or Tkinter for UI construction. In this work, we provide our own version of a Tkinter-based application with an embedded 2D Plane Wave Expansion Method (2D PWEM) with implemented diagonal anisotropy.

The functions that our application provides include photonic band computations in an oblique grid, the evaluation of iso-frequency contours for spatial dispersion control, and eigen-field computations given a specific symmetry of the PhC distribution. The application offers such functions for the diagonally anisotropic materials. Other graphic options of the User Interface include GIF generation and modern UI designs. The application also includes a function for importing custom-generated permittivity, allowing users to include their own custom permittivity distribution arrays along with the Brillouin zone of interest. Taking these points into account, we provide contributions to the field of computational photonics with our open-source UI:

- 2D PWEM method written in Python using customtkinter application design library, as seen in Fig 1. The greatest contribution presented here is the implemented models for isotropic and diagonally anisotropic materials.
- Cross-validation of already published results that encompass Photonic Crystal structures. Their respective properties, such as self-collimation and negative refractive index, are thoroughly studied.
- Numerical speed comparison study of 2D PWEM in Python and Matlab environments.
- Convergence study of the algorithm for high contrast dielectric Photonic Crystals.

## Numerical method

The ZenBand application is based on the Plane Wave Expansion Method, which is convenient to use when dealing with electromagnetic fields in periodic structures^27^. It is easy to implement and is less computationally intensive compared to other methods such as FDFD^28^. The algorithm, that is used in this application, was originally developed by dr. Raymond C. Rumpf^29^. The basic idea relies on Fourier transforming the Maxwell’s equations by expanding the electric, magnetic fields, dielectric permittivity and magnetic permeability in a plane wave basis. The set of equations is then rewritten in matrix form and an eigenvalue equation is constructed:

1 $$\begin{aligned} \left( \textrm{K}_{x} \llbracket \mu _{r, yy} \rrbracket ^{-1}\textrm{K}_{x} + \textrm{K}_{y}\llbracket \mu _{r, xx} \rrbracket ^{-1} \textrm{K}_{y} \right) s_{z}&= k_{0}^{2} \llbracket \varepsilon _{r, zz} \rrbracket s_{z},\end{aligned}$$

2 $$\begin{aligned} \left( \textrm{K}_{x} \llbracket \varepsilon _{r, yy} \rrbracket ^{-1} \textrm{K}_{x} + \textrm{K}_{y} \llbracket \varepsilon _{r, xx} \rrbracket ^{-1}\textrm{K}_{y} \right) u_{z}&= k_{0}^{2} \llbracket \mu _{r, zz} \rrbracket u_{z}; \end{aligned}$$

where $K_i$ is a diagonal wave vector matrix for *i*th component, $s_z$ is the electric field eigen-vector and $u_z$ is the magnetic field eigen-vector for *z* component, and double brackets denote convolution matirces. Here $k_0 = \omega / c_0$ is the eigenvalue. The equations become simpler for isotropic materials as permittivity and permeability convolution matrices for every component become the same (given $i=(x,y,z)$): $\llbracket \varepsilon _{r, ii} \rrbracket \rightarrow \llbracket \varepsilon _{r} \rrbracket$, $\llbracket \mu _{r, ii} \rrbracket \rightarrow \llbracket \mu _{r} \rrbracket$.

**Fig. 2:**
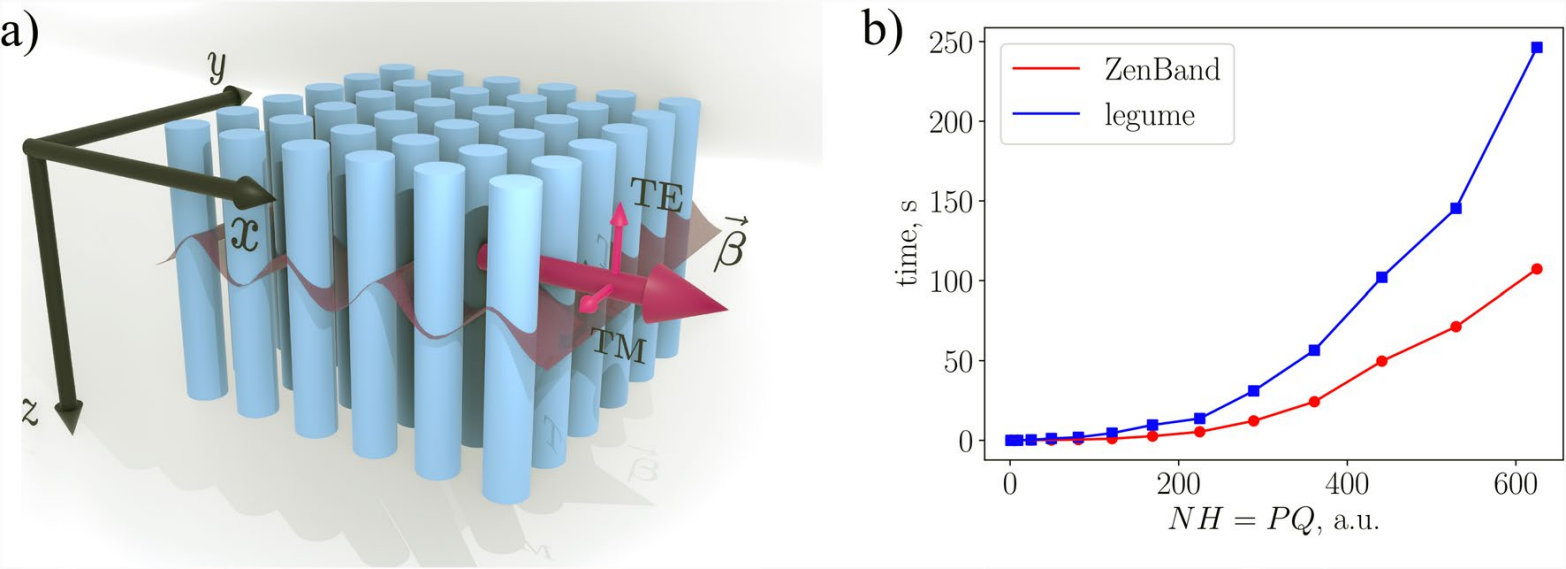
Simplified depiction of 2D PWEM concept (**a**). This example would be considered as TM polarization or E mode. Bloch wave vector $\vec {\beta }$ indicates the direction of wave propagation in a crystal. Graph (**b**) compares the time it takes to calculate band diagrams for a hexagonal lattice between legume^30^ and ZenBand. Letters *P* and *Q* indicate the number of spatial harmonics in *x* and *y* directions.

A schematic representation of the PWEM formalism is given in Fig. 2a. The blue columns symbolize a dielectric material that stretches to infinity in z direction. The red sinusoidal wave is a representation of a Bloch wave. The big red arrow depicts the Bloch wave vector ($\vec {\beta }$) and the two smaller arrows indicate the polarization convention. The polarization of the field is chosen by the component that oscillates in the *xy* plane. Two types of polarizations are present in the 2D model: Transverse Electric (TE) and Transverse Magnetic (TM) polarizations, which in the model are denoted as *H* and *E* modes, respectively. Such modes are the field components for which the PWEM problem is derived and solved, where the former field is $H_z(x,y)$ and the latter is $E_z(x,y)$.

A simplified 2D PWEM algorithm for the numerical method is presented in Algorithm 1. The process starts with the user defining the unit cell grid. If built-in lattice geometries are used, then direct and reciprocal lattice vectors are defined automatically. Next, convolution matrices are computed and an array of Bloch wave vectors (denoted as $\vec {\beta }$) is constructed. If either band diagrams or iso-frequency contours are calculated, a *for* loop is initialized. For every $\vec {\beta }$ array element, $K_i^{(n)}$ wave vector matrices are constructed (line 2), which are used to formulate the eigenvalue equations (lines 4–5 and 7–8). The equation that is depicted in line number 10 in Algorithm 1 can only solve for *z* component fields. If either $f_x$ or $f_y$ is required, additional equations have to be solved, which are presented in lines 13–14 and 16–17. The fields are then reshaped into 2D arrays (lines 17–18) and inverse Fourier transformed into their field distributions. 

**Algorithm 1:**
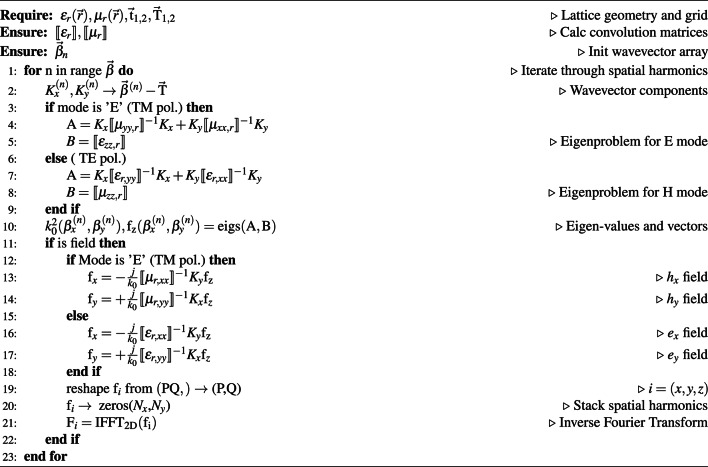
General 2D PWEM algorithm for photonic band analysis of diagonal anisotropy.

To provide further clarification , a simplified code example for field distribution calculations is provided in Listing 1. Every other function in ZenBand is implemented equivalently. The code starts by extracting parameters and initializing them. Then, the symmetry is defined by real and reciprocal lattice vectors, as well as an array of Bloch wave ($\vec {\beta }$) vectors, when calculating photonic bands. Afterwards, the device’s unit cell grid is created.

After properly defining the Bloch wave vector (i.e., the point in the reciprocal lattice), eigenvalue equations are constructed and solved for the chosen polarization, and eigen-vectors are returned. Since these vectors are in Fourier space, they must be reshaped into a 2D array and then inverse-transformed.

**Listing 1:**
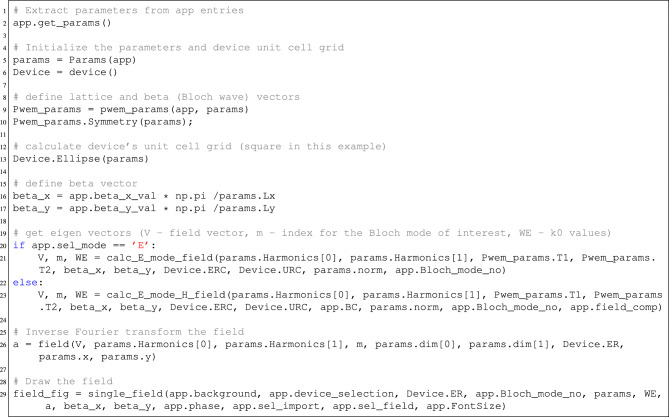
‘Calc Fz’ function handling of ZenBand.

The performance of ZenBand was tested against legume^30^—a Python package that includes a PWEM solver. Bands for a hexagonal lattice with $r^* = 0.2$ dielectric rods of $\varepsilon _r = 12$ were calculated using different numbers of spatial harmonics from $P = Q = 1$ up to $P = Q = 25$. An equal number of 60 $\vec {\beta }$ points were used in both cases. The time was measured from the point when the physical and numerical parameters are initialized up until the bands are computed. Figure 2b shows that ZenBand diverges more slowly compared to legume, when the number of spatial harmonics increases. A computer with a 12th Gen Intel(R) Core(TM) i3-1215U processor with 16 GB DDR4-3200 RAM was used for the simulations on Python 3.12.7. The maximum CPU frequency of 4.4 GHz could be achieved during runs.

## Results

### Application layout

**Fig. 3:**
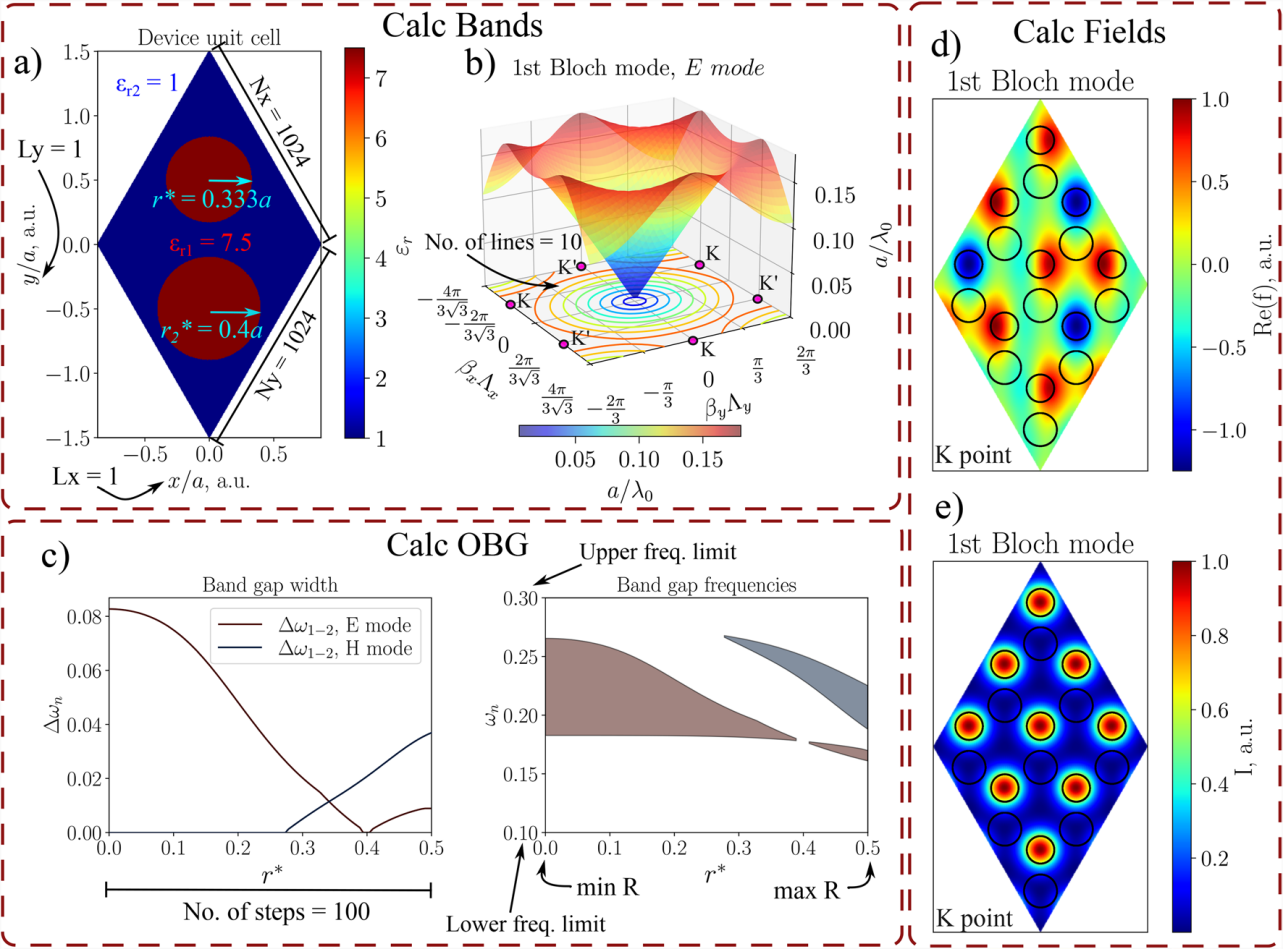
Different functions of ZenBand. The purpose of ’Calc Bands’ tab is to display the unit cell (**a**) and calculate photonic band diagrams, such as 3D iso-frequency contours (**b**). ’Calc OBG’ tab is used for omnidirectional band gap analysis—the output of ’Param sweep for R’ command is displayed in (**c**) where the value of $r^*$ was changed from 0 to 0.5*a* and $r_2^*$ was kept constant with a value of 0.4*a*. ’Calc Fields’ tab lets the user output field distributions of the device. Output of ’Calc Fz’ command is shown in (**d**) for the real part and (**e**) for the intensity of the field in K point in the reciprocal lattice.

The 2D PWEM application consists of three panels, as shown in Fig. 1. The first panel contains entries for PWEM parameters, the second panel features three tabs for executing the calculations, and the third panel provides additional options. PWEM parameters enable the user to conveniently adjust both the 2D device and computational performance. The program offers the freedom to choose between simple yet widely used PhC designs, including cylinders, frames, and rings in a rectangular grid, as well as cylinders in hexagonal and honeycomb grids. In addition, it also allows the user to change the number of periods for the unit cell in *x* and *y* directions, the width and eccentricity of the cylinders and the dielectric permittivity values. Computational performance can be adjusted by changing the unit cell grid size and the number of spatial harmonics used in *x* and *y* directions.

As mentioned earlier, the second panel is used to initiate the computation and is divided into three tabs. Each command, which is present in the second panel, returns a figure or creates a *.gif* file. If a figure is computed, the program creates a new top-level window containing the *matplotlib* figure and navigation toolbar, which allows the user to make simple manipulations and save the figure in a convenient format.

The first tab contains buttons which initialize band diagram and iso-frequency contour (which can be plotted as both 2D and 3D graphs, the latter of which is shown in Fig. 3b) calculations and hold entries for related parameters such as the number of beta vector points or the number of Bloch modes of interest. This tab also holds the ’Plot Device’ button, which returns a figure of the unit cell grid. To give an example, Fig. 3 will be used to display a portion of the functions and effects of different parameters. A honeycomb unit cell with different hole widths is chosen (Fig. 3a) replicating the results of a publication where the topology of 2D photonic crystals was investigated^31^. Lattices with such symmetry, known as valley photonic crystals, are a hot topic at the moment due to their excellent waveguide properties^32–35^.

**Fig. 4:**
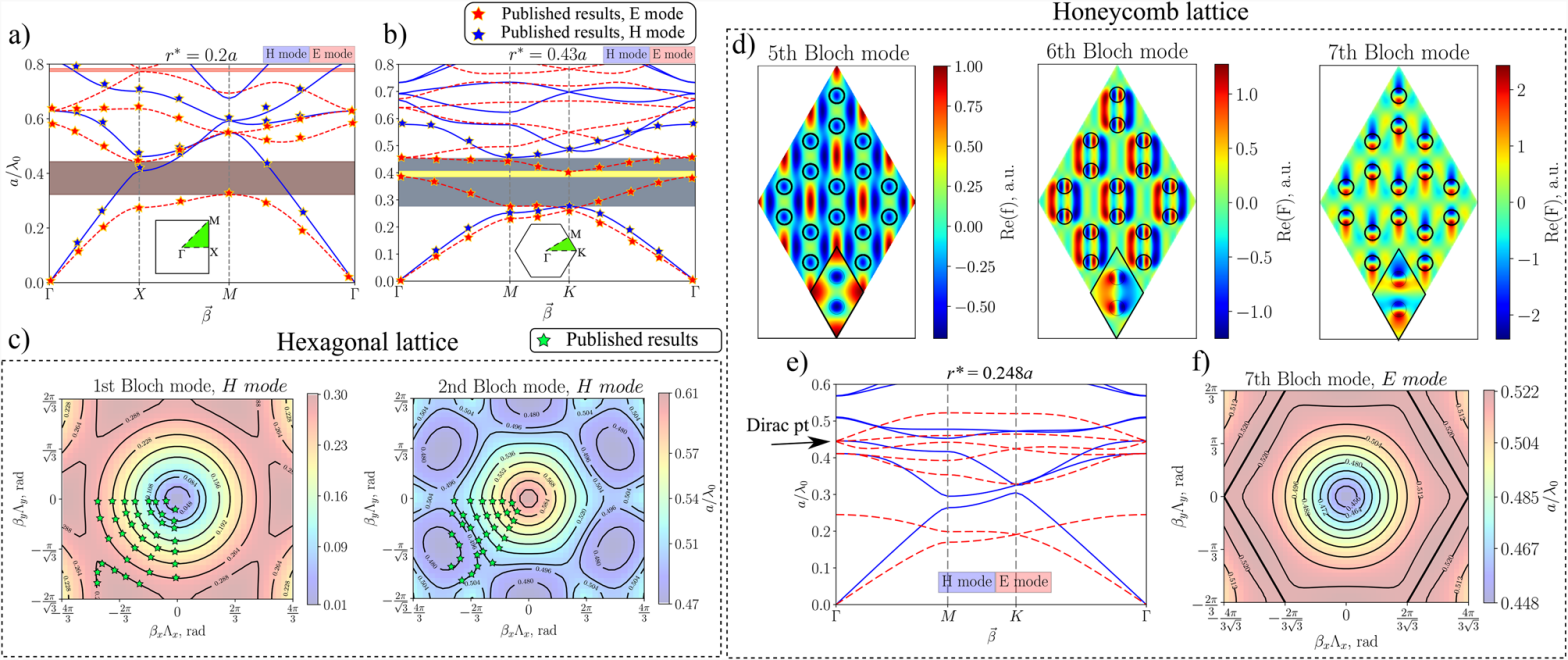
Cross-validation of the ZENBAND. The (**a,b**) parts compare band diagrams for square^36^ and hexagonal^37^ lattices, respectively. (**c**) Recreates iso-frequency contours from^38^. Field distributions for the honeycomb lattice, calculated with ZenBand, are shown in (**d**) and compared with^39^. (**e,f**) The band diagram with the Dirac point and iso-frequency contours from the publication.

The second tab was made with the omnidirectional bandgap (OBG) analysis in mind. The ’Calc Gap’ button returns a band diagram with highlighted bandwidths where OBGs are present. Gaps for E mode (TM pol.) are marked in red, gaps for H mode (TE pol.) are marked in blue, and complete photonic band gaps are colored yellow. This tab also includes commands for parameter sweep—the radius of the structure is increased in a desired interval and the change in OBG placement and bandwidth is plotted. The output of the function is demonstrated in Fig. 3c. The value of $r^*$ was increased from 0 to 0.5*a* while the radius of the other cylinder was kept constant at $r_2^* = 0.4a$. A similar concept is used to create a *.gif* file, which visualizes the change in the radius value within a unit cell and the evolution of the band diagram.

**Fig. 5:**
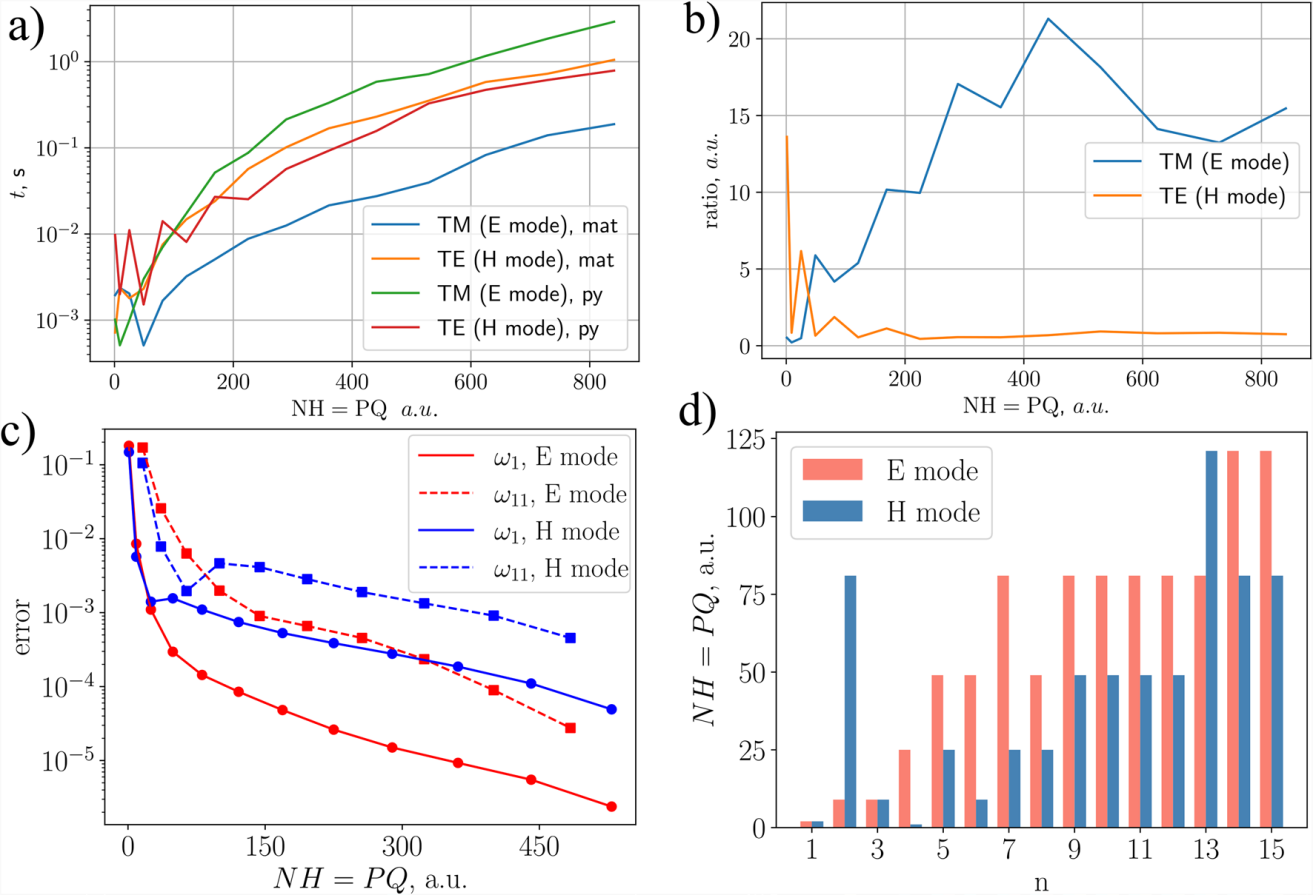
Direct time comparison (**a**) (in seconds) vs spatial harmonics, given P=Q. The ratio between Python numpy and MATLAB environments (**b**). The convergence test for a hexagonal lattice is depicted in graphs (**c,d**). Graph on the left (**c**) shows the error dependence on the number of spatial harmonics for the 1st and 11th bands for both E and H modes. The graph on the right (**d**) indicates the number of spatial harmonics needed to reach convergence for 1st–15th bands (denoted as *n* on the *x* axis). The convergence condition was set that the error must be lower than 0.01 %.

The third tab is dedicated to visualizing Bloch fields in a device. ’Get field gif’ command exhibits the evolution of a Bloch mode when the Bloch vector is being dragged across the perimeter of the irreducible Brillouin zone (IBZ). The other three buttons allow the user to visualize the Bloch mode at any point of the reciprocal lattice. When E mode is selected, ’Calc Fz’ returns a figure of the electric field *z* component in the device. ’Calc Fx’ and ’Calc Fy’ return fields of the magnetic field in the corresponding component. Figure 3d shows the distribution of the real part of the field, while (e) depicts the intensity distribution of the first Bloch mode in TM polarization at the K point of symmetry. Similar results can be found in “Figure 5" of the aforementioned publication^31^, where the field leaks deep into the crystal and is concentrated in the dielectric rods.

The third panel is dedicated to extra parameters and options. Since diagonally anisotropic materials can be adapted to the 2D PWEM algorithm, the option to analyze such materials was included. To enhance the functionality of this application, an option has been added to import data. By importing a .*mat* or .*npy* file with the device grid and necessary parameters, a user can compute band diagrams and field distributions for a wide variety of lattices with minimal limitations.

To enhance the user experience, an option to save parameters and enable different background modes was added. Since there are numerous adjustable parameters, saving them eliminates the need to fill them in every time a user wants to use the application, especially when only minor adjustments are desired. The background can also be changed between light and dark modes, providing the option to match the color themes of the graphs to one’s research and making the interface more pleasant to use in dark environments.

### Validation of results

To verify the validity of the application, results from other publications were replicated. In essence, the ZenBand solver can be divided into two types, even though they are based on the same set of equations—one for band diagrams (or iso-frequency contours) and one for field distributions. Another integral part of the program is geometry—square and hexagonal (oblique) symmetries require correctly defined lattice vectors in both real and reciprocal space.

Figure 4a compares ZenBand’s band diagram to a diagram that was published in literature^40^. A structure made of dielectric columns with radii of $r = 0.2 a$ and dielectric permittivity of $\varepsilon _r = 8.9$ was modeled in order to explain the appearance of band gaps in equivalent lattices. When the band gap widths for E mode were compared between ZenBand and the publication, only a $1.7 \%$ disagreement was found. This can be attributed to slightly different grid sizes of the unit cell or the number of spatial harmonics used in the calculations.

The validity of hexagonal symmetry in the application was verified by recreating a band diagram from a publication that investigated the possible uses of 2D PhCs for waveguides^37^. Once again, a band gap analysis for a dielectric waveguide with air holes was done ($r^* = 0.43a$, $\varepsilon _r = 11.6$, Fig. 4b). ZenBand results match the publication almost exactly in this case, there being no difference between the OBG width for E mode to the accuracy of the third digit after the decimal point and only a $1.6 \%$ disagreement for H mode.

Hexagonal symmetry was also inspected by repeating iso-frequency contour graphs that were taken from an article where an improvement in collimation effect for dielectric cylinders in a hexagonal lattice was demonstrated by breaking rotational symmetry^38^. The results were compared with symmetrical lattices ($r^* = 0.2a$, $\varepsilon _r = 9.8$) whose iso-frequency contours are depicted in Fig. 4c. Green stars represent contours in the publication that have the same frequency values as the ZenBand graph. It is clearly visible that the stars and contours overlap, providing nearly identical results.

To validate the field distribution capabilities of ZenBand, we investigated a honeycomb lattice beam splitter structure previously modeled using dielectric rods ^39^. The reference work specifies a normalized radius of $r^*_{\sqrt{3}} = 0.1433a$. It is important to note that this value implies a normalization factor of $\sqrt{3}$; for clarity and consistency, we define the standard radius as $r^* = 0.248a$ ($\approx 0.1433a \times \sqrt{3}$) and set $\varepsilon _r = 12$ within ZenBand.

Our calculations reveal a Dirac point consisting of three bands, spanning Bloch modes 5–7. Figure 4d compares the field distributions computed by ZenBand with the FEM-based results from COMSOL Multiphysics ^39^ (shown in the bottom panels). While the symmetries and dipole orientations align perfectly, some amplitude and phase discrepancies exist. These deviations are characteristic of the Plane Wave Expansion Method (PWEM) used in ZenBand.

Such a discrepancy is due to the nature of the inverse Fourier transform used in the model (see 21-line of Algorithm 1). The algorithm centers the Fourier field’s coefficients at the zeroes of a finite distribution of spatial harmonics, after which the inverse Fourier Transform is taken—this is technically called "zero-padding"^41^. Also, Gibbs^42,43^ phenomena occur near steep permittivity gradients, resulting in minor field distortions that do not affect the fundamental modal symmetries .

Figure 4e displays the recreated band diagram with the Dirac point at the $\Gamma$ point, while Fig. 4f illustrates the iso-frequency contours. Regarding frequency normalization, the reference work scales eigenvalues to frequency, whereas we utilize the normalized frequency ratio $a/\lambda _0$. We consider the latter more practical for geometric scaling in device design. Under this convention, our calculated Dirac point lies at $a/\lambda _0 = 0.4448$, corresponding well with the reference value of $a_{\sqrt{3}}/\lambda _0 = 0.4442$ (a deviation of $0.13\%$).

### Comparison between environments and convergence

The Fourier expansion-based algorithms and their optimized implementations ensure the fastest possible computation of eigenvalues and eigenvectors of a periodic system. This particularly involves the PWEM and Rigorous Coupled Wave Analysis (RCWA) algorithms for device scattering simulations^44^. The main result of the Fourier expansion of the fields is that the resolution of eigen-matrices depends only on the spatial harmonics used in the mentioned algorithms and thus can be implemented for low to medium contrasts of dielectric gratings, photonic crystals, etc. The outcome is increased computational speed and reduced memory requirements to perform tasks. Our goal is to compare and evaluate the performance of Python, MATLAB, and compiled C++ in terms of computing eigenvalue problems. The speed comparison (in seconds) is illustrated in Fig. 5a,b.

For implementation of the same algorithm in numpy, ordinary eigenvalue solver *np*.*linalg*.*eigs* outperforms Matlab for TE polarization (the H mode), because the permeability tensor is unity. In this case, Matlab performs 1.25–2 times slower starting from 225 harmonics (P=Q=15), where ratio$=t(\mathrm {Python)/t(\textrm{Matlab})}$ is used. However, the main issue here is the generalized eigenvalue solver, which is implemented using SciPy. It is built with the numpy library, rather than being compiled with C language. Unfortunately, it comes with a cost of computational speed, and thus underperforms compared to Matlab by a factor of 15–20 (see Fig. 5b) for E mode (TM polarization). The time capture is performed for a single $\beta$ iteration. The boost of performance can be obtained by solving such a generalized eigen-value problem either with a GPU (CuPy ), assuming more than $\mathrm {NH = 500}$ spatial harmonics are used, or if the spatial harmonic number is lower, then the Scipy’s linear algerba function ’eigh’ for generalized hermitian matrices can be utilized.

The performance of the algorithm was examined by studying the convergence. More precisely, the change in error was checked for different numbers of spatial harmonics. In this case, error is defined as:

3 $$\begin{aligned} error = \sqrt{\left( \frac{\omega ^{25\cdot 25}_n - \omega ^{P\cdot Q}_n}{\omega ^{25\cdot 25}_n} \right) ^2}, \end{aligned}$$

where $\omega ^{25x25}_n$ is the normalized frequency value for *n*th band with $P = Q = 25$ spatial harmonics (which could be considered highly accurate for circular patterns in a lattice) and $\omega ^{P \cdot Q}_n$ is the normalized frequency value for the same band with *P* and *Q* numbers of spatial harmonics in *x* and *y* directions.

The convergence test was performed on a hexagonal lattice with dielectric permittivity of $\varepsilon _r = 12$ and $r^* = 0.4 a$ radius air holes ($\varepsilon _r = 1$). The frequencies were analyzed at *K* point of the reciprocal lattice. The results are displayed in Fig. 5c,d. Graph (c) shows how the error changes for different numbers of spatial harmonics. Graph (d) shows how many harmonics are needed for different order bands (1st to 15th) to reach convergence, which is an error value less than $0.01 \%$.

It can be observed in Fig. 5c that the error reduces a bit faster for H mode when the number of harmonics is low ($<100$), but for a higher number of *PQ*, E mode starts to converge faster. This can be explained as follows. H mode does not require a generalized eigenvalue equation, and it converges slightly faster than E mode, but only up to a certain point. Once *PQ* reaches a large enough value, $\llbracket \varepsilon _{r} \rrbracket ^{-1}$ becomes increasingly more complex and induces more numerical errors. On the other hand, since the material is non-magnetic, $\llbracket \mu _{r} \rrbracket ^{-1}$ stays unity. The same idea can be used to explain Fig. 5d—the change of spatial harmonics for convergence is straightforward for E mode, as it increases with the number of the band. However, H mode has a less predictable dependence. Such dependence arises when the sum of two Fourier expansions that are discontinuous at the same point is multiplied together. Such a case can be found for the normal component of the electric field at the interface of a dielectric $\epsilon _r\cdot \vec {E}$. It is possible to handle such a problem, but it requires a normal vector matrix for each individual unit cell, which is a non-trivial task and would significantly impact the calculation speed. The method for such handling is abbreviated as Fast Fourier Factorization^45^.

**Fig. 6:**
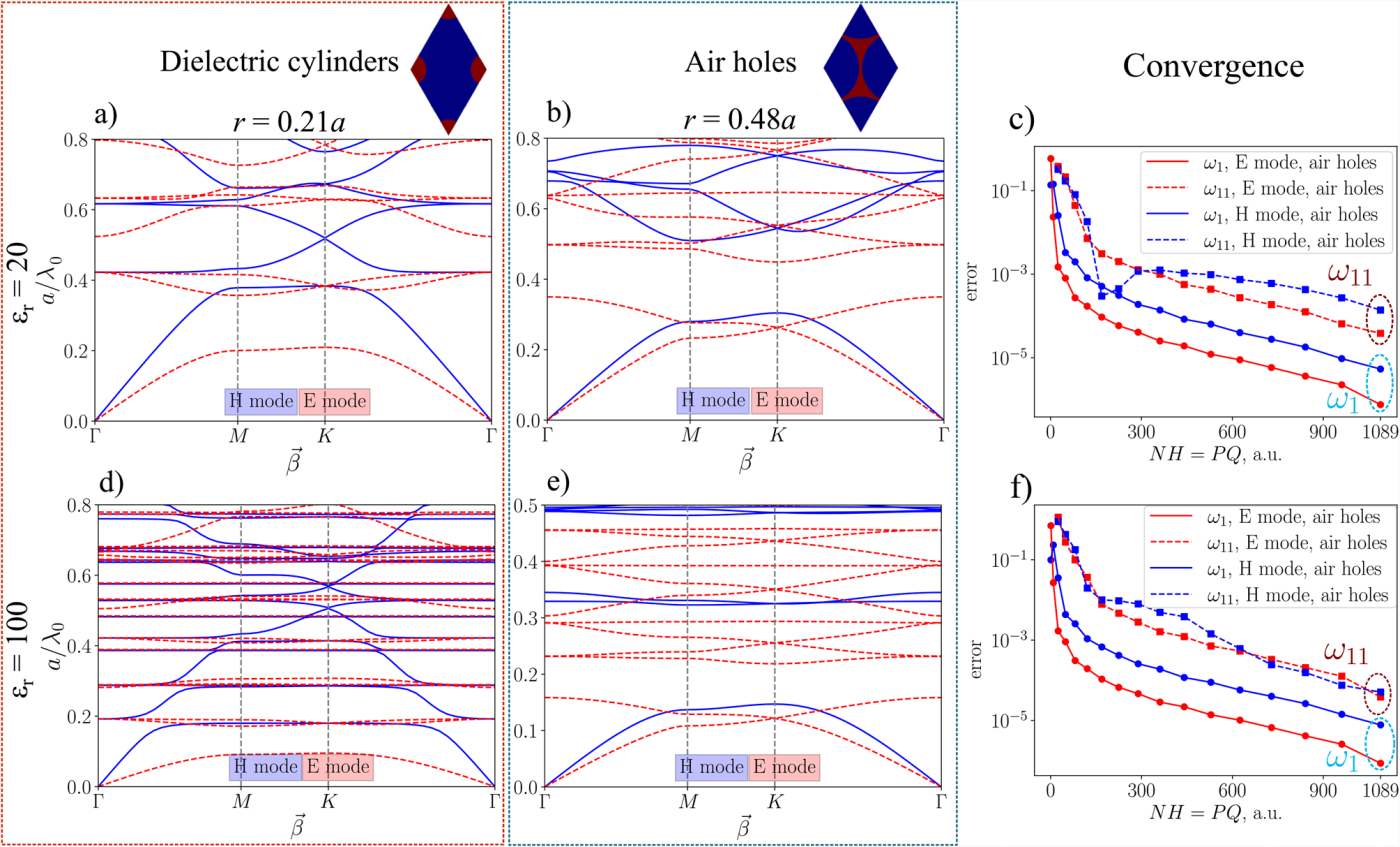
Convergence test under extreme conditions. (**a,b**) Depict dielectric cylinders and air holes as hexagonal arrays, with $\varepsilon _r = 20$. Graph (**c**) shows the convergence error of eigenvalue, based on (3) for 1st and 11th Bloch modes, including both polarizations. Graphs (**d,e**) are equivalent to (**a,b**) but the material’s relative permittivity $\varepsilon _r = 100$. Part (**f**) gives the convergence for the air hole distribution with the latter dielectric constant.

However, the PWEM must also be tested for extreme values of permittivity. The convergence test is presented in Fig. 6 for ZenBand’s eigenvalue solver under those extreme conditions. It was tested using dielectric rods in air and a dielectric slab with air holes in a hexagonal lattice. Two cases were analyzed: when relative dielectric permittivity is $\varepsilon _r = 20$ [graphs (a) and (b)] and $\varepsilon _r = 100$ [graphs (d) and (e)]. Figure 6c depicts the $\varepsilon _r = 20$ case for 1st and 11th Bloch modes, including both the TE and TM polarizations. Up to $\mathrm {NH =}33\cdot 33$ spatial harmonics were used to complete the simulation. The strong flattening of the photonic bands for E mode (cylinder case) and H mode (air hole case) specifies that their fields are strongly localized within the PhC structure. The strong localization of the $E_z$ field is within rods for high $\epsilon _r$, whereas for air hole distribution the strongest localization of the $H_z$ field is within the veins^40^.

## Conclusions

ZenBand is an open-source 2D PWEM solver with a simple graphical user interface that can be installed as a Python package. The program can be used to calculate photonic band diagrams and iso-frequency contours, analyze omnidirectional band gaps, and visualize Bloch fields. The application is easy to use, even for inexperienced or nonspecialized researchers, as it includes some of the most commonly found and widely applicable geometries. More experienced users have the opportunity to import their own devices and geometries if they desire to analyze more unusual or novel lattices.

Both the PWEM solver and the geometry of the lattices in ZenBand were validated by recreating graphs from various publications. The results are almost entirely consistent with other research, and the minor discrepancies can be attributed to numerical errors. The performance of the solver was also compared between Python and MATLAB environments. When the generalized eigenvalue solver is not used, Python performs exceptionally well and can be compared to MATLAB. Once the generalized eigenvalue solver is used, performance drops significantly; however, it remains sufficiently fast and outperforms other open-source solvers.

## Data Availability

The datasets used and/or analyzed during the current study are available from the corresponding author on reasonable request.
